# Subarachnoid Hemorrhage Revealing a Spinal Dural Arterio-Venous Fistula: About a Rare Complication

**DOI:** 10.5334/jbsr.3219

**Published:** 2023-08-09

**Authors:** Ibtissam El Ouali, Salma El Hous, Hassan Nouali

**Affiliations:** 1 Resident in radiology department of military hospital of Rabat, Morocco; 2 Professor in radiology department of military hospital of Rabat, Morocco

**Keywords:** Subarachnoid hemorrhage, spinal dural arteriovenous fistula, MRI

## Abstract

**Teaching Point:** Subarachnoid hemorrhage is a rare complication of spinal dural arteriovenous fistulas (dAVF) with very characteristic imaging features, knowledge of which is crucial for positive diagnosis and rapid management.

## Case History

**Figure 1:**
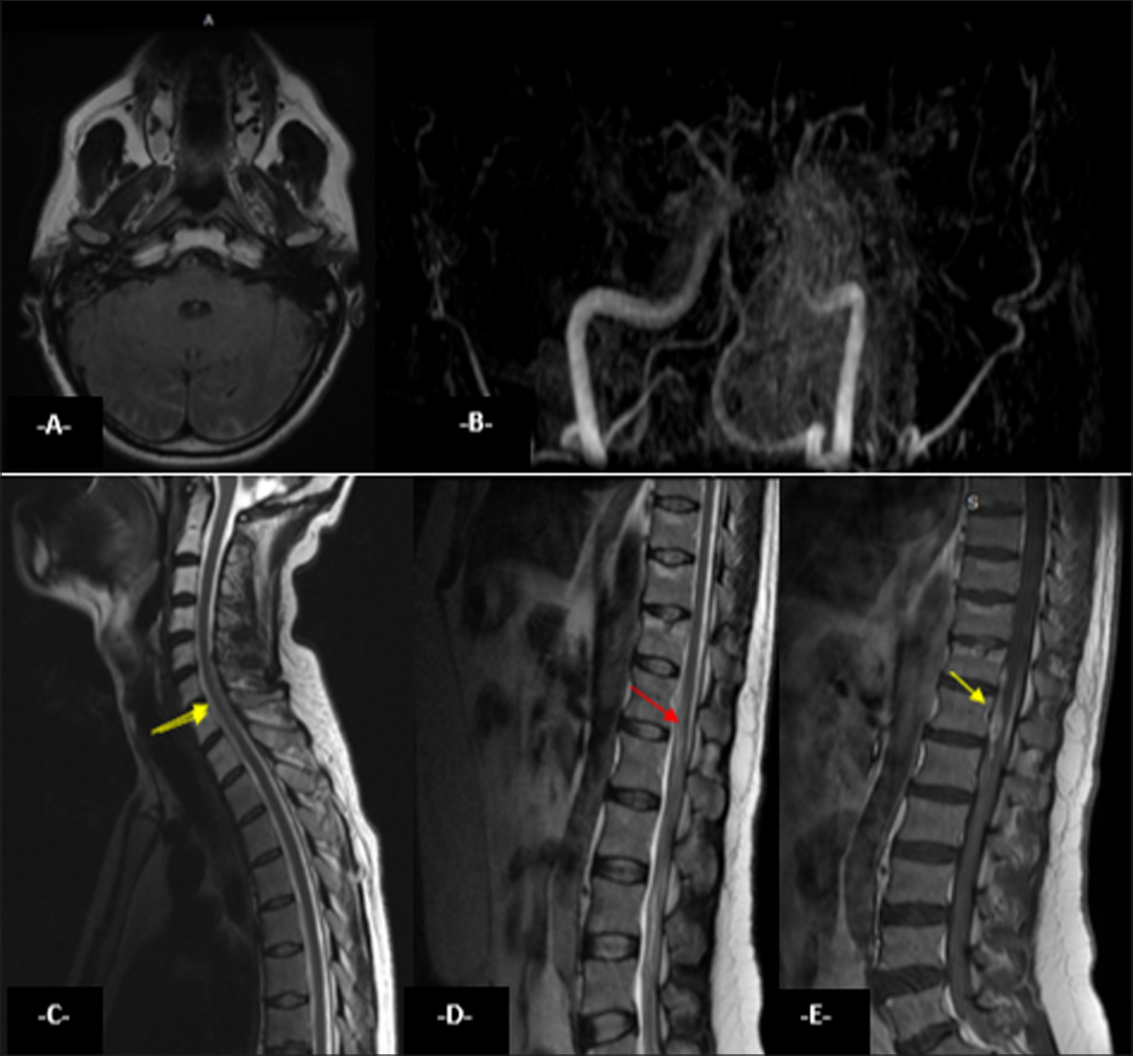
Axial magnetic resonance imaging of the brain showing hyperintensity in the subarachnoid space on T2 FLAIR **(A)** with a normal Time Of Flight (TOF) angiography. Sagittal magnetic resonance imaging with T2 **(C,D)** and T1 **(E)** spin echo (TSE) sequences reveals intradural and epidural hemorrhage (yellow arrows) and hypersignal of the spinal cord from T11 to L1 of the terminal cone (red arrow).

We report the case of 45-year-old male without a known history of neurological disease, presenting progressive weakness of lower limbs and tingling-like paresthesia. The evolution of the disease is marked by the onset of thunderclap headache. An endocranial computed tomography (CT) performed in the emergency department showed no particular anomaly. Then, a cerebral magnetic resonance imaging (MRI) was performed showing on axial images hyperintensity in the subarachnoid compatible with a peri mesencephalic subarachnoid hemorrhage space on T2 FLAIR with a normal time of flight (TOF) angiography (Figure 1A).

Cerebral angiography showed no specific findings.

A medullar MRI was then suggested and demonstrated on T2-sagittal MRI (Figure 1C–D, red arrow) intramedullary hyperintensity (edema) involving the conus medullaris and T1 sequences reveals epidural hyperintense collection of the spinal cord from T11 to L1 of the terminal cone compatible with epidural hematoma and subarachnoid hemorrhage (Figure 1E, yellow arrows).

**Figure 2:**
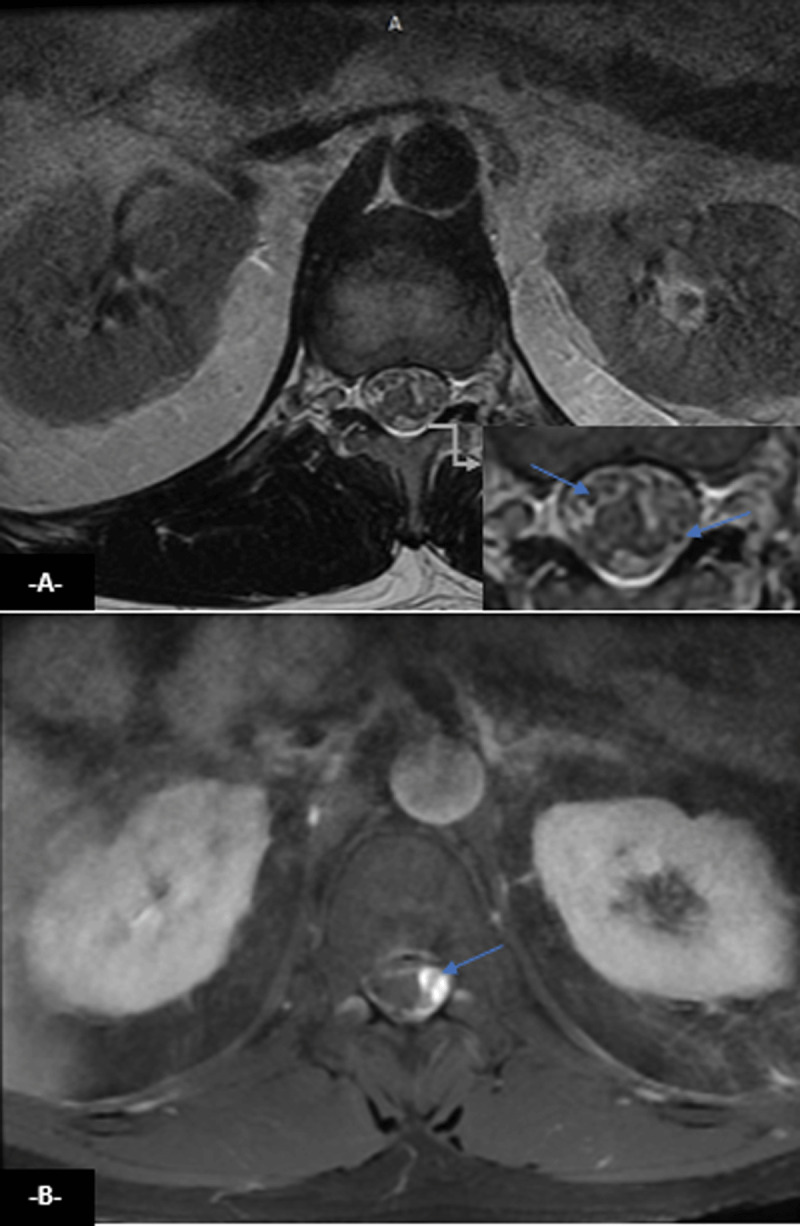
Medullar axial T2 **(A)** and post-enhanced T1 **(B)** images shows flow voids lateral to spinal cord with a serpentine enhancing veins on the cord surface (arrows).

Also, it showed on axial T2 (Figure 2A) and post-enhanced T1 (Figure 2B) images Intradural extramedullary flow voids lateral to spinal cord with a serpentine enhancing veins on the cord surface, T2 hypointense in the periphery of the cord enhancing on T1 post contrast axial images (Figure 2, arrows).

Patient was put under surveillance with symptomatic treatment and showed a recovered partial recovery of his motricity and sensitivity.

## Comments

Spinal dural arteriovenous fistulas (dAVF) are the most common spinal vascular malformations (75 to 80%) and more frequently affect male subjects over 50 years that exhibits arteriovenous shunting at the level of the internal leaflet dura mater, corresponding to an abnormal connection between the arterial supply from the medullary dura mater and the medullary venous system and no exact etiology has been fully elucidated [1].

The clinical presentation of sdAVF is often progressive and non-specific mimicking demyelinating or degenerative diseases of the spine, which explains a frequent delay in diagnosis, resulting in an irreversible damage despite an endovascular or surgical intervention.

Hemorrhage is very rare but occasionally encountered and may be a source of unexplained subarachnoid hemorrhage.

MRI shows an association of centrally located hyperintense signal on T2-weighted images on TSE sequence, corresponding to chronic hypoxic congestive myelopathy attributable to venous hyper pressure hindering venous circulation, dilated peri medullary vessels, hypointense ‘flow void’ phenomena hypo intense in T2w images, appearing as enlarged and serpiginous veins anteriorly and/or posteriorly to the cord, enhancing after administration of gadolinium The absence of dilated peri medullary vessels do not formally eliminate the diagnosis of Davf. Dynamic MRA (angio-MRI) sequences are more sensitive assets to detect this abnormality.

Principal differential diagnosis on imaging includes intramedullary neoplasm, spinal arteriovenous malformation where hemorrhage is common with acute onset, affecting males and females equally.

Our case is unique as it depicts the importance of including spinal cord malformations in unexplained cerebral sub arachnoid hemorrhages.
